# Effects of 6 Months of Active Commuting and Leisure-Time Exercise on Fibrin Turnover in Sedentary Individuals with Overweight and Obesity: A Randomised Controlled Trial

**DOI:** 10.1155/2018/7140754

**Published:** 2018-05-16

**Authors:** Anne Sofie Gram, Martin Bæk Petersen, Jonas Salling Quist, Mads Rosenkilde, Bente Stallknecht, Else-Marie Bladbjerg

**Affiliations:** ^1^Department of Biomedical Sciences, Faculty of Health and Medical Sciences, University of Copenhagen, Copenhagen, Denmark; ^2^Unit for Thrombosis Research, Department of Regional Health Research, University of Southern Denmark, Odense, Denmark; ^3^Department of Clinical Biochemistry, Hospital of South West Jutland, Esbjerg, Denmark

## Abstract

Obesity and exercise constitute important factors for cardiovascular disease risk, but the long-term effects of different exercise modalities on haemostatic biomarkers are not well elucidated. We investigated the effects of 6 months of active commuting or leisure-time exercise on measures of fibrin turnover in individuals who are overweight and obese. Ninety younger (20–40 years), sedentary, healthy women and men who are overweight and obese (BMI: 25–35 kg/m^2^) were randomised to 6 months of habitual lifestyle (CON, *n*=16), active commuting (BIKE, *n*=19), or leisure-time exercise of moderate (MOD, ∼50% VO_2_peak reserve, *n*=31) or vigorous intensity (VIG, ∼70% VO_2_peak reserve, *n*=24). Fasting blood samples (baseline and 3 and 6 months) were analysed for cholesterols and triglycerides, thrombin generation, prothrombin fragment 1 + 2, D-dimer, fibrin clot properties, and fibrinolytic activity. We observed no differences between CON, BIKE, MOD, and VIG during the intervention and no time effects for any of the variables measured despite increased VO_2_peak in all exercise groups. We found no difference between CON and all exercise groups combined and no gender-specific effects of exercise. Our findings suggest that thrombin generation capacity, coagulation activation, fibrin clot structure, and lysability are unaffected by long-term active commuting and leisure-time exercise in women and men who are overweight and obese.

## 1. Introduction

Cardiovascular disease (CVD) has a well-known association with obesity and physical inactivity, and it is well accepted that regular exercise reduces the overall risk of CVD [1–4]. Nevertheless, the Look AHEAD trial suggested that an intensive lifestyle intervention through caloric restriction and increased physical activity does not reduce the rate of cardiovascular events among adults with type 2 diabetes [5]. However, a recent post hoc analysis of the Look AHEAD trial revealed a reduced risk of cardiovascular mortality after increased physical activity or weight reduction [6].

From a public health perspective, exercise is a time-consuming factor in everyday life. As recently suggested by Andersen [7] and supported by longitudinal and cross-sectional data, active commuting may be an easy and effective way to increase daily activity levels [3, 8–11]. Interestingly, a meta-analysis by Hamer and Chida [12] found a protective effect of active commuting on cardiovascular outcomes (mortality, myocardial infarction, coronary heart disease, hypertension, and stroke), which was more profound among women than men. In the CARDIA study, active commuting was associated with increased cardiorespiratory fitness in both women and men and a reduced risk of obesity and CVD risk factors in men, although not in women [13]. However, there is still a lack of long-term randomised controlled trials investigating whether active commuting and leisure-time exercise of different intensities can reduce the risk of CVD to the same extent.

Randomised controlled trials in healthy individuals assessing CVD incidence as a primary endpoint are practically infeasible, but intermediate CVD risk markers can instead be studied. Thrombosis is a key factor in CVD caused by disturbances in the haemostatic balance [14–18], and numerous studies have tried to elucidate the effects of exercise on the haemostatic balance [19]. In a recent study [20], we observed that daily endurance exercise at vigorous intensity for 3 months in overweight men exerts an effect on blood coagulation in the direction of anticoagulation, expressed as reduced endogenous thrombin potential (ETP). Others have similarly reported that short-term (<3 months) aerobic exercise affects the balance between blood coagulation and fibrinolysis [21–25], but the results are conflicting and may depend on the duration and intensity of the prescribed exercise regimens. No studies have investigated the effects of active commuting on the haemostatic balance.

Therefore, the present randomised controlled study is aimed at determining the effects of 6 months of active commuting or leisure-time exercise at moderate or vigorous intensity on markers of coagulation activation, fibrin clot properties, and fibrinolytic activity in women and men who are overweight and obese (class 1), that is, participants with a high potential of benefit in terms of CVD prevention.

## 2. Materials and Methods

### 2.1. Participants and Study Design

The project GO-ACTIWE (Governing Obesity: Active Commuting To Improve health and Wellbeing in Everyday life, http://go.ku.dk/) is a randomised controlled trial addressing the health effects of physical activity in adults who are overweight and obese (class 1), and the participants and study design have been described in detail elsewhere [26, 27]. All procedures performed in this study were in accordance with the ethical standards of the ethics committee of the Capital Region of Denmark (H-4-2013-108) and with the Helsinki Declaration. The trial is registered at http://clinicaltrial.gov (ID-code: NCT01962259). Recruitment and data collection took place between October 2013 and June 2016. Informed consent (written and oral) was obtained from all participants included in the study.

In brief, we randomised 130 healthy (blood pressure < 140/90 mmHg; fasting blood glucose < 6.1 mmol/l; no regular use of medication), sedentary (regular exercise < 2 hours/week and active commuting <5 km/day; VO_2_peak: women < 40 ml O_2_/kg/min and men < 45 ml O_2_/kg/min), young (20–45 years), nonsmoking, Caucasian women and men who are overweight/obese (class 1) (BMI 25–35 kg/m^2^; fat percentage: women ≥ 32% and men ≥ 25%). Exclusion criteria included abnormal resting electrocardiogram and parents or siblings with type 2 diabetes, and for women, they include postmenopause, pregnancy, or planned pregnancy [26, 27].

Participants were stratified for gender and randomly allocated in a 1 : 2 : 2 : 2 manner to maintain either their habitual lifestyle in a control group (CON, *n*=18), active commuting by bike (BIKE, self-selected intensity, *n*=35), moderate-intensity leisure-time endurance exercise (MOD, 50% VO_2_peak reserve, *n*=39), or vigorous-intensity leisure-time endurance exercise (VIG, 70% VO_2_peak reserve, *n*=38). Active commuting or leisure-time endurance exercise was prescribed 5 days/week, and the total weekly exercise dose prescribed in all three exercise groups was 1600 kcal in women and 2100 kcal in men. The intervention period was 6 months, and the exercise intervention was monitored with the use of heart rate (HR) monitors individually adjusted after 6 weeks and 3 months based on changes in maximal HR, VO_2_peak, and body weight. Exercise intensity for MOD and VIG was calculated from the maximal oxygen uptake reserve method [28]. To avoid potential carry-over effects and preanalytic disturbances, all participants were instructed not to exercise one day prior to testing. To avoid seasonal variation, the number of participants was evenly distributed throughout the year. Dietary intake was ad libitum throughout the intervention, and participants were advised to maintain their habitual diet [26, 27]. Self-reported energy intake and macronutrient intake have been published elsewhere and did not change in any of the intervention groups [29].

To facilitate exercise adherence, participants in all three exercise groups were instructed to upload training data every week and were in frequent contact (E-mail, telephone, and text messages) with the research staff. Adherence to the exercise intervention from baseline till 6 months was calculated as exercise-induced energy expenditure (% of the energy expended during the intervention in proportion to the prescribed energy expenditure) and exercise intensity (% of VO_2_peak reserve in proportion to the prescribed intensity), respectively. Completers demonstrated an excellent exercise energy expenditure adherence (>90% in BIKE, MOD, and VIG) and intensity adherence (BIKE: self-selected intensity of 54%; MOD: 49%; VIG: 66%) to the intervention from baseline till follow-up, and aerobic capacity (VO_2_peak) increased during the intervention in all exercise groups compared with CON [26].

**Figure 1:**
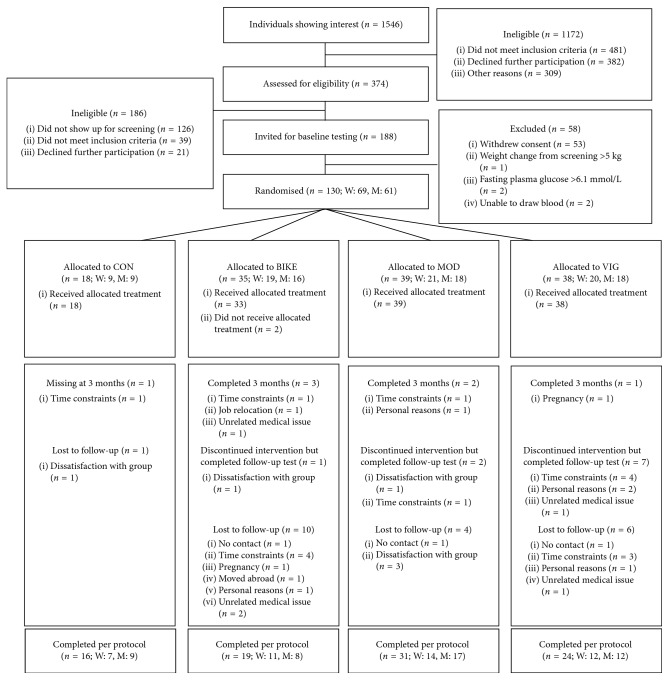
Flow of the progress of GO-ACTIWE.

In total, 90 participants completed the study, and the participant flow, including the number of completers, reasons for dropouts, and participants available for analysis, including gender distribution, is displayed in Figure 1. The population characteristics are described in Table 1.

### 2.2. Blood Sampling

Blood samples were obtained at baseline and at 3 and 6 months between 8.30 and 9.30 am after an overnight fast (≥10.5 h) including abstention from alcohol on the day before testing. Blood samples were collected with minimal stasis by the BD Vacutainer system (Becton Dickinson, Plymouth, UK) after 20 minutes of rest in the supine position. Blood samples were collected in lithium heparin tubes (BD Ref.: 368884) and trisodium citrate tubes (0.109 M Na_3_citrate, BD Ref.: 363048). Platelet-poor plasma was prepared by centrifugation of lithium heparin tubes for 10 min at 2100 xg (4°C) and citrate tubes for 20 min at 2000 xg (20°C) immediately after blood sampling. Plasma was transferred to 500 *µ*l aliquots and stored at −80°C.

### 2.3. Blood Analyses

Lithium heparin samples were analysed in one batch for triglycerides and HDL, LDL, and total cholesterol concentrations with absorption photometry (Roche Cobas 8000 c702 module).

Citrated plasma samples were rapidly thawed in a water bath at 37°C and analysed in one series for each participant. All technicians were blinded to the study groups. The calibrated automated thrombogram method was applied to assess plasma thrombin generation (Thrombinoscope BV, Maastricht, Netherlands) [30, 31]. The thrombin generation was performed by mixing 80 *µ*l plasma with 20 *µ*l fluorogenic substrate-calcium chloride (FluCa) and 20 *µ*l trigger reagent with a final concentration of 5 pM tissue factor (TF) and 4 *µ*M phospholipids. Fluorescence was read in a Fluoroskan Ascent microplate fluorometer (Fisher Scientific, Slangerup, Denmark) with a 390/460 nm filter set. Thrombin generation curves were generated with the Thrombinoscope software (Thrombinoscope BV, Maastricht, Netherlands) to display the lag time, time to peak, peak, start tail, and ETP. Velocity index was defined as the peak divided by the difference between the time to peak and lag time.

A commercial ELISA method (Enzygnost F1 + 2; Siemens, Marburg, Germany) using mouse monoclonal antibodies was used to measure concentrations of prothrombin fragment 1 + 2 (F1 + 2) in plasma. Concentrations of D-dimer were measured by an immunoturbidimetric method (STA-Liatest D-DI; Diagnostica Stago, Asniéres-sur-Seine, France).

Global fibrinolytic activity in plasma was determined by a fibrin plate assay [32]. The fibrinolytic activity was calibrated against the 3rd International Standard for recombinant t-PA (NIBSC 98/714). The fibrin clot properties were studied using turbidity measurements [33–35]. Briefly, plasma was mixed with thrombin (final concentration 0.11 IU/ml) and CaCl_2_, with and without addition of rt-PA, and turbidity was recorded for 30 min as optical density (OD) at 405 nm. The maximal turbidity increment (*V*_max_) and fibrin clot lysis were calculated as previously reported [33]. Next, the fibrin clot structure was determined by measuring the OD at 405, 540, 608, and 690 nm after an overnight incubation, and the fiber mass-length ratio, fiber diameter, and fiber mass density were calculated [33].

### 2.4. Statistics

To ensure adequate power (>80%), the sample size was determined for the primary outcome, ETP, based on results from a previous study [26]. The calculations suggested inclusion of 140 participants in total: 40 participants in each exercise group and 20 in the control group [27]. The present analysis of GO-ACTIWE is an efficacy analysis with the aim to determine biological effects of long-term exercise, and data were analysed as observed with a per-protocol analysis.

Baseline values were compared between the four groups using one-way analysis of variance (ANOVA), and for the primary effect variable, completers and noncompleters were compared using a *t*-test. To determine differences between the four groups and account for the multiple time points in the study design (baseline and 3 and 6 months), a mixed between-within subjects ANOVA was performed. If no significant group x time interaction was observed, main effects of time and between-group effects were reported. The mixed between-within subjects ANOVA was adjusted for relevant confounders (lipids, ETP, time to peak, peak, lag time, and start tail were adjusted for baseline values of BMI).

Supplementary analyses were conducted to determine potential gender-specific effects of exercise. In these analyses, all exercise groups (BIKE, MOD, and VIG) were pooled to enable determination of differences in exercise effects between women and men. Also, the combined exercise groups (*n*=74) were compared with the control group to examine the effects of exercise, irrespective of the type of exercise.

Non-normally distributed data (triglycerides, ETP, lag time, F1 + 2, D-dimer, and fibrinolytic activity) were logarithmically transformed. Data are presented as mean (95% CI) or geometric mean (geometric 95% CI). *p* < 0.05 was considered significant. Data were analysed with IBM SPSS Statistics 23.0 (IBM Corp., Armonk, NY, USA).

## 3. Results

Concentrations of lipids at baseline and at 3 and 6 months are presented in Table 2, and measures of the thrombin generation test (start tail, time to peak, peak, ETP, lag time, velocity index, F1 + 2, D-dimer, and fibrinolytic activity) and measures of the fibrin clot structure (*V*_max_, clot lysis, fiber mass-length ratio, fiber diameter, and fiber mass density) are presented in Tables 3 and 4. There were no differences between the groups at baseline for any of the variables measured. Also, baseline values of the primary effect variable ETP did not differ between completers (1960 (1885; 2037) nM·min) and noncompleters (1901 (1803; 2004) nM·min) (*p*=0.375).

We observed no differences between the four groups during 6 months of intervention. Thus, there were no significant interactions between the group and time, no significant main effects of time, and no significant between-group effects as presented in Tables 2–4.

The gender-specific analysis did not reveal interactions between gender and time for total cholesterol (*p*=0.268), LDL cholesterol (*p*=0.236), HDL cholesterol (*p*=0.380), triglycerides (*p*=0.438), ETP (*p*=0.246), time to peak (*p*=0.171), start tail (*p*=0.349), peak (*p*=0.174), lag time (*p*=0.533), velocity index (*p*=0.231), F1 + 2 (*p*=0.399), D-dimer (*p*=0.891), and fibrinolytic activity (*p*=0.396), or for the measures of the fibrin structure *V*_max_ (*p*=0.116), clot lysis (*p*=0.563), fiber mass-length ratio (*p*=0.148), fiber diameter (*p*=0.128), and fiber mass density (*p*=0.714). Furthermore, there were no main effects of time and no between-gender effects for any of the variables measured except that women had higher concentrations of HDL cholesterol (*p* < 0.0005) and lower concentrations of triglycerides (*p* < 0.01) than men (data not shown).

When the three exercise groups were combined and compared with the control group, we observed no interactions between the group and time for total cholesterol (*p*=0.508), LDL cholesterol (*p*=0.518), HDL cholesterol (*p*=0.263), triglycerides (*p*=0.593), ETP (*p*=0.688), time to peak (*p*=0.655), start tail (*p*=0.769), peak (*p*=0.449), lag time (*p*=0.889), velocity index (*p*=0.678), F1 + 2 (*p*=0.430), D-dimer (*p*=0.611), fibrinolytic activity (*p*=0.977), *V*_max_ (*p*=0.340), clot lysis (*p*=0.637), fiber mass-length ratio (*p*=0.897), fiber diameter (*p*=0.271), and fiber mass density (*p*=0.507). Furthermore, there were no main effects of time and no between-group effects for any of the variables measured (data not shown).

## 4. Discussion

The major finding in the present study of women and men who are overweight and obese was that 6 months of active commuting or leisure-time endurance exercise at two different intensities had no effects on the thrombin generation potential, markers of coagulation activation, measures of fibrin clot properties, and fibrinolytic activity in plasma. Also, no effects were observed for triglycerides and cholesterols.

No previous long-term (>3 months) exercise studies have investigated the effects of active commuting and leisure-time exercise on markers of the haemostatic balance in healthy, younger women and men who are overweight and obese. In the present study, the exercise intervention was very carefully controlled to ensure correct energy expenditure, the frequency of exercise, and exercise intensity. This was achieved by monitoring exercise HR and via frequent contact between the staff and participants. The success of the exercise intervention is supported by the observed increase in cardiorespiratory fitness in all exercise groups (Table 1). Furthermore, preanalytical factors potentially affecting coagulation activation *in vitro* were meticulously controlled for during blood sampling and handling.

The thrombin generation test measures the capacity of plasma to form thrombin. High levels of ETP have been associated with thromboembolism and arterial vascular disease [36, 37]. In a previous study, we observed that ETP was lowered by daily vigorous-intensity endurance exercise at high and moderate doses for 3 months among healthy men who are overweight [20]. This was not confirmed in the present study, where no effects on ETP were observed after 3 and 6 months of active commuting or moderate- or vigorous-intensity leisure-time exercise 4-5 times/week. To our knowledge, only Hilberg et al. have previously investigated the effects of physical training on fasting levels of ETP in healthy adults, and similar to the present study, they reported no effects of 12 weeks of vigorous-intensity exercise on men who are overweight and who exercised with a frequency of 3-4 times/week [24].

In the present study, no effects of active commuting or leisure-time exercise were observed on F1 + 2, D-dimer, markers of coagulation activation, and cardiovascular risk [38]. Hilberg et al. [24] also demonstrated unchanged levels of F1 + 2 following 12 weeks of vigorous-intensity exercise in men (40–60 y), whereas Lockard et al. [39] showed a decrease in F1 + 2 following 6 months of vigorous-intensity aerobic exercise (3 sessions/week) in combination with the American Heart Association diet in men and postmenopausal women (50–75 y). The deviating findings may be ascribed to the age difference between the populations and the differences in dietary habits. Thus, Lockard et al. [39] demonstrated that exercise combined with a healthy diet is an excellent primary prevention remedy in an older population at higher risk of developing CVD compared to the younger and healthier population in our study, where we solely intervened on exercise behavior. In accordance with our findings, results from the Look AHEAD study [40] showed no changes in D-dimer in individuals with type 2 diabetes and in individuals who are obese after a one-year randomised trial on intensive lifestyle intervention including exercise and a low-calorie diet.

Active commuting and leisure-time exercise did also not affect the haemostatic balance in the direction of fibrinolysis measured as global fibrinolytic activity and fibrin clot lysis. Furthermore, fibrin clot formation (*V*_max_) and fibrin fiber structure (fiber mass-length ratio, fiber diameter, and fiber mass density) did not differ between the groups and did not change over time. To our knowledge, our study is the first long-term exercise trial focusing on measures of fibrin clot properties. Fibrin clots composed of compact networks with thin fibers are more resistant to lysis and are associated with CVD [41, 42]. Fibrin clot characteristics can be improved by smoking cessation, medications (e.g., antidiabetics, coagulant therapy, and statins) [41, 42], and oral contraceptives [35]. We demonstrate, however, that long-term exercise does not add to the list of modifiers of clot formation, fiber properties, and clot lysis.

The lack of effect on clot lysability is supported by the results for fibrinolytic activity measured by the fibrin plate assay. The plasma fibrinolytic activity is primarily determined by the active forms of tissue plasminogen activator (t-PA) and its inhibitor plasminogen activator inhibitor type 1 (PAI-1), and no previous studies have assessed the global fibrinolytic activity in relation to long-term exercise. Only a few studies have measured t-PA activity and PAI-1 activity with either no changes observed after 12 weeks of vigorous-intensity exercise in moderately overweight men [24] or favorable effects on t-PA activity (increase) and PAI activity (reduction) after long-term (6–12 months) vigorous-intensity exercise 3–5 days/week in older, but not younger, men [43] and in men with peripheral arterial disease [44]. Also, the protein concentration of PAI-1 (PAI : Ag) affects the global fibrinolytic activity, and we have recently demonstrated that active commuting and leisure-time exercise have no effects on PAI : Ag in the present study [26], thus supporting the results for global fibrinolytic activity.

We did not also observe any effects on triglycerides and HDL, LDL, or total cholesterol levels, except minor differences in concentrations between women and men. The lack of effect may imply that the participants were too healthy at study inclusion to obtain effects on blood lipids following exercise.

It is unclear why we were unable to reproduce our previous results on increased exercise and reduced ETP, but one explanation could be that the exercise frequency was reduced from 6-7 sessions/week in our previous study [20] to 4-5 sessions/week in the present study. Acute exercise is known to activate coagulation and fibrinolytic properties [22, 24, 45–47], and the findings of the present study, along with the findings by Hilberg et al. [24], may suggest that less-frequent activation of coagulation through ∼4 exercise sessions/week for 3 or 6 months does not lower ETP levels in overweight individuals. We speculate that high-frequency (daily) acute exercise with coagulation activation might consume coagulation factors and thereby reduce ETP. This is supported by Huskens et al. who reported a reduction in ETP after acute exercise [48]. In the present study, the participants were instructed not to exercise one day prior to testing in order to avoid potential carry-over effects of coagulation activation and possibly also on ETP. Exercise intensity does not explain the deviation since the participants in the VIG group in the present study exercised at the same intensity as in our previous study [20].

In the statistical analysis of ETP, we adjusted for BMI due to a significant correlation between these two variables. It is possible that a greater weight loss is necessary to affect ETP in the direction of anticoagulation, and participants in our previous study [20] had greater reductions in BMI than in the present study. Also, we included both women and men in the present study, and one could speculate that there is a gender-specific response to exercise with respect to the thrombin generation potential. In terms of effects of exercise on body weight and composition, several studies have suggested that men experience greater weight loss, body fat loss, and increase in fat-free mass compared to women [10, 49, 50]. Furthermore, 44 women completed the present study, of which 27 (CON: *n*=5; BIKE: *n*=8; MOD: *n*=10; and VIG: *n*=4) used oral contraceptives throughout the study. Oral contraceptives are known to have procoagulant properties [51], but it is uncertain whether oral contraceptives modify the effect of exercise on coagulation activation. However, the subanalyses in the present study did not reveal any gender-specific effects of exercise for any of the variables measured. The study was, however, not powered to reveal gender-specific effects [26, 27].

Besides the many reported study strengths, the study also has limitations. The power calculations suggested that 140 participants should be included in order to detect significant effects of exercise on ETP. We managed to randomise 130 participants, and due to a variety of reasons (Figure 1), dropout rates in BIKE and VIG were higher than the expected 20%. As a consequence, it cannot be excluded that the lack of significant effects may be due to type II errors, although the same number of participants as in VIG revealed an effect of vigorous exercise training on ETP in our previous study of only men [20], and in the present study, there was not even a trend towards a decrease in ETP. The sample size was, however, large enough to demonstrate an effect on other markers associated with CVD, that is, an increase in cardiorespiratory fitness (VO_2_peak) in all the three exercise groups compared with CON and a decrease in the inflammatory marker C-reactive protein in BIKE and MOD [26], whereas we observed no effects on the classical blood lipid risk markers. Furthermore, when the exercise groups were combined into one large group and compared with the control group, we also did not observe any differences between exercise and control groups.

In conclusion, our study suggests that 6 months of active commuting and leisure-time exercise at different intensities do not have notable effects on markers of coagulation activation, fibrin clot structure, and lysability in healthy, younger women and men who are overweight and obese (class 1).

## Figures and Tables

**Table 1:** Maximal aerobic capacity and body mass index at baseline and at 3 and 6 months.

	CON (*n*=16)	BIKE (*n*=19)	MOD (*n*=31)	VIG (*n*=24)
Age (years)	35 (31; 39)	35 (32; 39)	33 (30; 35)	36 (34; 40)
VO_2_peak (ml/min)				
Baseline	2792 (2330; 3253)	2678 (2452; 2905)	2700 (2501; 2900)	2684 (2414; 2954)
3 months	2823 (2377; 3269)^a^	3023 (2737; 3310)^∗^^#^	2884 (2657; 3110)^∗^	3025 (2729; 3321)^∗^^#^
6 months	2770 (2319; 3220)	2975 (2688; 3262)^∗^^#^	2926 (2699; 3154)^∗^^#^	3068 (2770; 3366)^∗^^#^
BMI (kg/m^2^)				
Baseline	30.2 (28.9; 31.5)	30.4 (28.8; 32.0)	29.2 (28.5; 29.9)	30.1 (29.2; 30.5)
3 months	30.8 (29.3; 32.3)	30.0 (28.4; 31.6)^#^	29.1 (28.3; 29.9)	29.5 (28.5; 30.5)^∗^^#^
6 months	30.9 (29.3; 32.5)	29.8 (28.2; 31.4)^#^	28.9 (28.1; 29.8)	29.3 (28.1; 30.4)^∗^^#^

Data are mean (95% CI). ^a^*n*=14; ^#^significantly different from CON (ANCOVA adjusted for baseline values); ^∗^significant within the group change from baseline (repeated measures ANOVA); subjects' characteristics were previously reported by Gram et al. [26]; CON, control group; BIKE, active commuting exercise group; MOD, moderate-intensity leisure-time exercise group; VIG, vigorous-intensity leisure-time exercise group; BMI, body mass index.

**Table 2:** Concentrations of blood lipids at baseline and at 3 and 6 months.

	CON (*n*=16)	*n* ^b^	BIKE (*n*=19)	*n* ^b^	MOD (*n*=31)	*n* ^b^	VIG (*n*=24)	*p* values
Cholesterol (mM)								0.833^c^, 0.518^d^, 0.179^e^
Baseline	4.50 (4.15; 4.86)	14	4.83 (4.43; 5.23)	18	4.60 (4.33; 4.86)	28	5.02 (4.59; 5.44)
3 months	4.50 (4.00; 5.00)	14	4.87 (4.55; 5.18)	18	4.59 (4.30; 4.88)	28	4.93 (4.48; 5.37)
6 months	4.31 (4.02; 4.60)	14	4.86 (4.42; 5.30)	18	4.51 (4.27; 4.75)	28	4.93 (4.49; 5.38)

LDL cholesterol (mM)								0.832^c^, 0.870^d^, 0.573^e^
Baseline	2.83 (2.53; 3.12)	14	3.00 (2.62; 3.39)	18	2.83 (2.57; 3.09)	28	3.03 (2.71; 3.34)
3 months	2.78 (2.39; 3.17)	14	3.10 (2.79; 3.41)	18	2.81 (2.59; 3.03)	28	3.08 (2.73; 3.42)
6 months	2.67 (2.40; 2.94)	14	3.07 (2.68; 3.45)	18	2.84 (2.63; 3.05)	28	2.98 (2.58; 3.38)

HDL cholesterol (mM)								0.621^c^, 0.428^d^, 0.359^e^
Baseline	1.18 (1.03; 1.33)	14	1.23 (1.11; 1.35)	18	1.23 (1.09; 1.38)	28	1.31 (1.12; 1.49)
3 months	1.17 (1.00; 1.33)	14	1.31 (1.15; 1.48)	18	1.27 (1.44; 1.40)	28	1.34 (1.16; 1.51)
6 months	1.12 (1.00; 1.23)	14	1.30 (1.15; 1.45)	18	1.28 (1.14; 1.42)	28	1.32 (1.14; 1.50)

Triglycerides (nM)^a^								0.143^c^, 0.244^d^, 0.848^e^
Baseline	1.05 (0.79; 1.38)	14	1.15 (0.88; 1.51)	18	1.25 (1.02; 1.54)	28	1.13 (0.80; 1.57)
3 months	1.03 (0.70; 1.52)	14	1.09 (0.87; 1.37)	18	1.23 (0.98; 1.54)	28	1.09 (0.85; 1.41)
6 months	1.09 (0.82; 1.44)	14	1.10 (0.84; 1.44)	18	1.02 (0.85; 1.23)	28	1.19 (0.88; 1.60)

Data are unadjusted mean (95% CI) or ^a^geometric mean (geometric 95% CI); ^b^number of samples available for analysis; ^c^*p* values for interactions between the group and time; ^d^*p* values for main effects of time for all groups combined; ^e^*p* values for between-group effects; CON, control group; BIKE, active commuting exercise group; MOD, moderate-intensity leisure-time exercise group; VIG, vigorous-intensity leisure-time exercise group; LDL, low-density lipoprotein; HDL, high-density lipoprotein.

**Table 3:** Biomarkers of coagulation activation at baseline and at 3 and 6 months.

	CON (*n*=16)	*n* ^b^	BIKE (*n*=19)	*n* ^b^	MOD (*n*=31)	*n* ^b^	VIG (*n*=24)	*n* ^b^	*p* values
ETP (nM·min)^a^									0.314^c^, 0.413^d^, 0.555^e^
Baseline	1985 (1819; 2166)	16	2049 (1803; 2328)	18	1975 (1859; 2097)	31	1883 (1750; 2026)	22
3 months	2000 (1844; 2171)	15	2143 (1957; 2346)	16	1963 (1832; 2103)	29	1970 (1849; 2094)	24
6 months	2078 (1907; 2358)	16	2108 (1884; 2358)	16	1965 (1844; 2094)	31	2008 (1858; 2126)	24

Time to peak (min)									0.882^c^, 0.942^d^, 0.453^e^
Baseline	6.78 (6.26; 7.33)	16	6.46 (5.78; 7.15)	18	6.36 (5.97; 6.75)	30	6.87 (6.23; 7.51)	23
3 months	6.59 (5.97; 7.21)	15	6.24 (5.70; 6.79)	16	6.34 (5.93; 6.34)	29	6.85 (6.28; 7.44)	24
6 months	6.37 (5.66; 7.05)	16	6.28 (5.67; 6.89)	16	6.26 (5.84; 6.68)	31	6.57 (5.96; 7.19)	24

Start tail (min)^a^									0.934^c^, 0.490^d^, 0.993^e^
Baseline	26.7 (25.5; 27.9)	16	26.9 (25.6; 28.2)	18	26.5 (25.7; 27.2)	31	26.6 (25.5; 27.5)	22
3 months	27.0 (26.7; 28.4)	15	27.5 (26.3; 28.6)	16	27.0 (25.9; 28.1)	29	26.8 (25.7; 27.9)	24
6 months	26.9 (25.5; 28.9)	16	27.2 (25.8; 28.6)	16	27.5 (25.8; 21.2)	31	27.1 (25.9; 28.1)	24

Peak (nM)									0.550^c^, 0.245^d^, 0.522^e^
Baseline	301 (264; 339)	16	309 (263; 354)	18	307 (285; 330)	30	283 (256; 313)	22
3 months	306 (274; 338)	15	327 (288; 365)	16	299 (273; 325)	29	289 (263; 314)	24
6 months	329 (289; 368)	16	321 (276; 367)	16	300 (271; 328)	31	303 (278; 327)	24

Lag time (min)^a^									0.727^c^, 0.845^d^, 0.799^e^
Baseline	3.40 (3.11; 3.71)	16	3.17 (2.87; 3.49)	18	3.26 (3.00; 3.53)	30	3.29 (2.91; 3.72)	22
3 months	3.39 (2.85; 3.44)	15	3.13 (2.85; 3.42)	16	3.16 (2.91; 3.45)	29	3.34 (2.98; 3.75)	24
6 months	3.26 (2.97; 3.58)	16	3.15 (2.82; 3.53)	16	3.15 (2.86; 3.27)	31	3.25 (2.92; 3.62)	24

Velocity index (nM/min)									0.398^c^, 0.155^d^, 0.372^e^
Baseline	99 (82; 116)	16	107 (79; 134)	18	111 (96; 127)	30	91 (74; 106)	22
3 months	106 (89; 122)	15	114 (92; 135)	16	109 (91; 128)	29	91 (76; 105)	24
6 months	112 (86; 138)	16	124 (88; 160)	16	104 (90; 118)	31	103 (88; 117)	24

F1 + 2 (pmol/l)^a^									0.474^c^, 0.611^d^, 0.758^e^
Baseline	196 (149; 260)	16	212 (184; 245)	17	212 (184; 245)	30	174 (138; 218)	22
3 months	207 (169; 254)	14	198 (164; 240)	16	198 (164; 240)	30	207 (161; 265)	24
6 months	174 (140; 216)	16	223 (184; 271)	16	223 (184; 271)	30	182 (149; 222)	23

D-dimer (*µ*g/l)^a^									0.622^c^, 0.439^d^, 0.795^e^
Baseline	0.26 (0.21; 0.32)	16	0.31 (0.25; 0.39)	18	0.29 (0.25; 0.34)	30	0.26 (0.21; 0.30)	23
3 months	0.27 (0.21; 0.35)	15	0.28 (0.21; 0.34)	16	0.27 (0.23; 0.31)	30	0.30 (0.25; 0.37)	24
6 months	0.23 (0.19; 0.29)	16	0.29 (0.23; 0.32)	18	0.27 (0.23; 0.31)	31	0.25 (0.21; 0.30)	24

Data are unadjusted mean (95% CI) or ^a^geometric mean (geometric 95% CI); ^b^number of samples available for analysis; ^c^*p* values for interactions between the group and time; ^d^*p* values for main effects of time for all groups combined; ^e^*p* values for between-group effects; CON, control group; BIKE, active commuting exercise group; MOD, moderate-intensity leisure-time exercise group; VIG, vigorous-intensity leisure-time exercise group; BMI, body mass index; ETP, endogenous thrombin potential; F1 + 2, prothrombin fragment 1 + 2.

**Table 4:** Measures of the fibrin clot structure and fibrinolytic activity at baseline and at 3 and 6 months.

	CON (*n*=16)	*n* ^b^	BIKE (*n*=19)	*n* ^b^	MOD (*n*=31)	*n* ^b^	VIG (*n*=24)	*n* ^b^	*p* values
*V*_max_ (OD/min)									0.189^c^, 0.495^d^, 0.567^e^
Baseline	0.80 (0.72; 0.89)	15	0.76 (0.70; 0.83)	15	0.86 (0.77; 0.95)	28	0.76 (0.69; 0.84)	22
3 months	0.80 (0.72; 0.88)	15	0.76 (0.69; 0.83)	15	0.80 (0.72; 0.88)	28	0.75 (0.68; 0.81)	22
6 months	0.83 (0.74; 0.91)	15	0.76 (0.65; 0.87)	15	0.77 (0.69; 0.85)	28	0.77 (0.70; 0.83)	22

Clot lysis (%)									0.714^c^, 0.476^d^, 0.061^e^
Baseline	38.0 (29.5; 46.4)	15	37.5 (31.2; 43.8)	15	44.4 (39.5; 49.2)	28	38.9 (33.5; 44.3)	22
3 months	36.9 (28.8; 45.1)	15	37.7 (30.5; 44.8)	15	48.1 (42.3; 54.0)	28	39.1 (32.8; 45.3)	22
6 months	36.3 (27.7; 44.8)	15	35.4 (28.0; 42.7)	15	45.2 (40.0; 50.7)	28	39.3 (32.4; 46.2)	22

Fiber mass-length ratio (×10^12^ Da/cm)									0.292^c^, 0.501^d^, 0.788^e^
Baseline	6.76 (6.18; 7.34)	15	7.05 (6.57; 7.54)	15	7.19 (6.73; 7.64)	28	6.86 (6.35; 7.36)	22
3 months	6.85 (6.46; 7.23)	15	7.04 (6.45; 7.63)	15	6.96 (6.50; 7.43)	28	7.02 (6.42; 7.63)	22
6 months	6.87 (6.18; 7.56)	15	7.26 (6.24; 8.28)	15	6.85 (6.50; 7.21)	28	7.49 (6.80; 8.18)	22

Fiber diameter (*µ*m)									0.117^c^, 0.883^d^, 0.761^e^
Baseline	0.14 (0.13; 0.15)	15	0.14 (0.13; 0.15)	15	0.14 (0.14; 0.15)	28	0.14 (0.13; 0.15)	22
3 months	0.14 (0.14; 0.15)	15	0.14 (0.13; 0.15)	15	0.14 (0.13; 0.14)	28	0.14 (0.13; 0.15)	22
6 months	0.14 (0.14; 0.15)	15	0.13 (0.12; 0.14)	15	0.14 (0.13; 0.14)	28	0.14 (0.13; 0.15)	22

Fiber mass density (×10^22^ Da/cm^3^)									0.476^c^, 0.414^d^, 0.466^e^
Baseline	4.53 (4.20; 4.85)	15	5.01 (4.06; 5.97)	15	4.54 (4.33; 4.74)	28	4.54 (4.31; 4.76)	22
3 months	4.33 (4.14; 4.52)	15	4.47 (4.25; 4.69)	15	4.68 (4.43; 4.92)	28	4.62 (4.40; 4.85)	22
6 months	4.30 (4.06; 4.54)	15	5.28 (4.36; 6.20)	15	4.66 (4.48; 4.85)	28	4.75 (4.30; 5.19)	22

Fibrinolytic activity (IU/ml)^a^									0.878^c^, 0.960^d^, 0.123^e^
Baseline	78 (52; 117)	15	113 (73; 175)	15	71 (56; 91)	29	94 (69; 130)	22
3 months	76 (54; 106)	15	115 (81; 163)	15	73 (55; 96)	29	93 (69; 124)	22
6 months	78 (54; 112)	15	94 (64; 137)	15	76 (61; 95)	29	99 (73; 133)	22

Data are unadjusted mean (95% CI) or ^a^geometric mean (geometric 95% CI); ^b^number of samples available for analysis; ^c^*p* values for interactions between the group and time; ^d^*p* values for main effects of time for all groups combined; ^e^*p* values for between-group effects; CON, control group; BIKE, active commuting exercise group; MOD, moderate-intensity leisure-time exercise group; VIG, vigorous-intensity leisure-time exercise group; OD, optical density.
